# Global transcriptome analysis of two wild relatives of peanut under drought and fungi infection

**DOI:** 10.1186/1471-2164-13-387

**Published:** 2012-08-13

**Authors:** Patricia M Guimarães, Ana CM Brasileiro, Carolina V Morgante, Andressa CQ Martins, Georgios Pappas, Orzenil B Silva, Roberto Togawa, Soraya CM Leal-Bertioli, Ana CG Araujo, Marcio C Moretzsohn, David J Bertioli

**Affiliations:** 1 EMBRAPA Recursos Genéticos e Biotecnologia. Parque Estação Biológica, CP 02372 Final W5 Norte, Brasília, DF, Brazil; 2 EMBRAPA Semi –Árido, BR 428, Km 152, Zona Rural - CP 23, Petrolina, PE, Brazil; 3 Universidade de Brasília, Campus I, Brasília, DF, Brazil; 4 Universidade Católica de Brasília, Campus II, 916 Norte, Brasília, DF, Brazil

## Abstract

**Background:**

Cultivated peanut (*Arachis hypogaea*) is one of the most widely grown grain legumes in the world, being valued for its high protein and unsaturated oil contents. Worldwide, the major constraints to peanut production are drought and fungal diseases. Wild Arachis species, which are exclusively South American in origin, have high genetic diversity and have been selected during evolution in a range of environments and biotic stresses, constituting a rich source of allele diversity. *Arachis stenosperma* harbors resistances to a number of pests, including fungal diseases, whilst *A. duranensis* has shown improved tolerance to water limited stress. In this study, these species were used for the creation of an extensive databank of wild Arachis transcripts under stress which will constitute a rich source for gene discovery and molecular markers development.

**Results:**

Transcriptome analysis of cDNA collections from *A. stenosperma* challenged with *Cercosporidium personatum* (Berk. and M.A. Curtis) Deighton**,** and *A. duranensis* submitted to gradual water limited stress was conducted using 454 GS FLX Titanium generating a total of 7.4 x 10^5^ raw sequence reads covering 211 Mbp of both genomes. High quality reads were assembled to 7,723 contigs for *A. stenosperma* and 12,792 for *A. duranensis* and functional annotation indicated that 95% of the contigs in both species could be appointed to GO annotation categories. A number of transcription factors families and defense related genes were identified in both species. Additionally, the expression of five *A. stenosperma* Resistance Gene Analogs (RGAs) and four retrotransposon (FIDEL-related) sequences were analyzed by qRT-PCR. This data set was used to design a total of 2,325 EST-SSRs, of which a subset of 584 amplified in both species and 214 were shown to be polymorphic using ePCR.

**Conclusions:**

This study comprises one of the largest unigene dataset for wild Arachis species and will help to elucidate genes involved in responses to biological processes such as fungal diseases and water limited stress. Moreover, it will also facilitate basic and applied research on the genetics of peanut through the development of new molecular markers and the study of adaptive variation across the genus.

## Background

Legumes are an important source of protein for humans and livestock. Cultivated peanut (*Arachis hypogaea*) is one of the most widely grown grain legumes in the world. It is widely cultivated mainly in Asia, Africa and the Americas, and is valued for its high protein and unsaturated oil contents [1,2]. Worldwide, the major constraints to peanut production are drought and fungal diseases including Early (ELS) and Late Leaf Spots (LLS), the latter caused by *Cercosporidium personatum*[3-5].

Wild *Arachis* species, which are exclusively South American in origin, have high genetic diversity and have been selected during evolution in a range of environments and biotic stresses, and constitute a rich source of allele diversity [6-8]. The species *A. stenosperma* harbors resistances to a number of pests, including the root-knot nematode *Meloidogyne* spp. [9] and fungal diseases [10,11], whilst *A. duranensis* is originated from regions with relatively low rainfall [12]. These wild relatives are a rich source of new alleles for peanut improvement as they have sufficient polymorphism for their genetic characterization, and the tracking of genome segments that confer these resistances during introgression into cultivated peanut [13-15]. Both species have been exploited as a resource for gene discovery, interpretation of genomic sequences and marker development [11,15-19] and are also parents of a recently developed RIL (Recombinant Inbred Line) diploid mapping population.

In recent years, a relatively large number of EST sequences have been made available in the National Center for Biotechnology Information (NCBI) public database for *A. hypogaea* (151,352). However, fewer resources exist for *A. duranensis* (35,292) and *A. stenosperma* (6,264). In addition, a whole genome sequencing project for peanut (tetraploid) remains a challenge, in part due to the size of the genomes compared to model plants, even for the diploid species (*A. duranensis* 1,260 Mbp vs. 115 Mbp in *Arabidopsis thaliana*), but even more because of the high repetitive DNA content [20,21].

Functional genomics studies, using microarrays and subtractive libraries (SSH), identified genes potentially associated to stress responses to *C. personatum* and drought stress in *Arachis* spp. [3,4,22,23]. However, to our knowledge, no massal transcriptome analysis in stressed wild Arachis is available.

Increased transcriptome sequence resources should facilitate basic and applied research on genetics, contribute to the development of molecular markers, facilitate comparative genomics and aid in the study of adaptive variation across the genus. In addition, transcriptome data can assist on the elucidation of genes involved in biological processes, such as defense responses to biotic and abiotic stress, which Transcription Factors (TFs) are notably associated [24,25], and have hardly been studied in wild Arachis.

Although it is still a challenge to assemble a new whole complex genome using Next-Generation Sequencing technologies (NGS) (454/Illumina), the smaller size and reduced repetitive content of the transcriptome together with increased coverage facilitates the *de novo* transcriptome assembly using these technologies [26]. Next-generation sequencing technologies have facilitated large scale generation of ESTs cost-effectively, and allowed the whole transcriptome analysis of a number of smaller scale legume crops such as chickpea, lentil, mungbean and pigeonpea [27-30]. Moreover, deep sequencing has enabled the identification of new transcripts not present in previous model crops EST collections, such as *Arabidopsis*[31] and rice [32], and the massive identification of molecular markers such as SNPs (Single Nucleotide Polymorphisms) and SSRs (Simple Sequence Repeats) [33,34]. In addition, EST-SSRs being genic in origin, frequently display a high degree of transferability between related species.

In the present study 743,232 sequence reads were produced using Roche/454 GS FLX Titanium, generating a total of 17,912 unigenes for *A. stenosperma* and 21,714 for *A. duranensis* submitted to infection with *C. personatum* and gradual water limited stress, respectively. Contigs derived from these reads were annotated into functional categories separately for each species, and the expression of five Arachis RGAs (Resistance Gene Analogs) and four sequences related to the only fully-characterized Arachis retrotransposon (FIDEL) [20] further analyzed by qRT-PCR (quantitative reverse transcription-PCR). This database was also used to design a set of 214 EST-SSRs primers which showed polymorphism via electronic PCR (ePCR) between the two species studied.

The genomic resources developed in this study can, in association with other tools already developed for wild Arachis, contribute to accelerate genetics and breeding of peanut and contribute particularly for the elucidation of genes involved in responses to important biological processes such as fungal diseases and water limited stress in peanut and other legumes.

## Results

### EST sequencing and assembly

**Figure 1:**
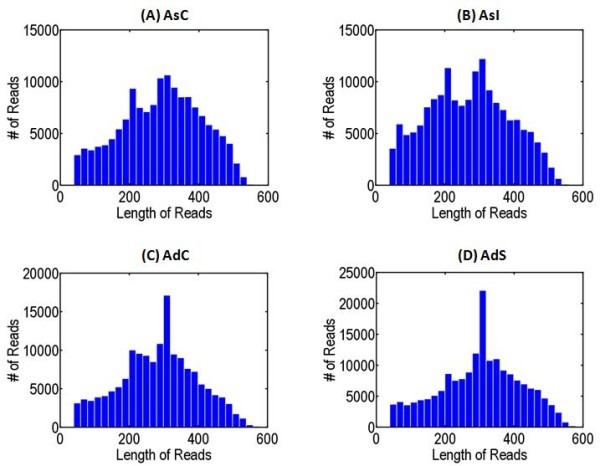
**454 GS FLEX Titanium read length distribution for each library (bp).** AsI- *A. stenosperma* inoculated with *C. personatum*; AsC- *A. stenosperma* control; AdS- *A. duranensis* under water limited stress; AdC- *A.duranensis* control.

A total of 7.4 x 10^5^ raw sequence reads covering 211 Mbp were generated in a single 454 GS FLX Titanium run (Table 1) on the four libraries constructed from two *Arachis* species subjected to biotic (*A. stenosperma*/*C. personatum*) and abiotic (*A. duranensis*/water limitation) stress and respective controls. After eliminating adapter sequences, low quality chromatograms and “masking” unwanted sequences (rDNA, mitochondrial, repetitive) a total of 3.1 x 10^5^ processed high quality reads were obtained for *A. stenosperma* and 2.7 x 10^5^ for *A. duranensis* (Table 1). The average length of high quality sequence reads was 278 bp for *A. stenosperma* and 282 bp for *A. duranensis*, enabling coverage of 85 and 78 Mbp of the genomes, respectively (Figure 1, Table 1).

**Table 1:** Total number and length of 454 GS FLX Titanium reads for each library

**Libraries**	**Number of total reads**	**Total length (bp)**	**Number of high quality reads**	**High quality length (bp)**
*A. stenosperma*	362,631	100,394,273	310,430	85,649,830
AsI	194,076	51,609,348	162,393	42,868,986
AsC	168,555	48,784,925	148,037	42,780,844
*A. duranensis*	380,601	111,425,609	276,884	78,147,055
AdS	200,031	60,268,742	138,275	39,660,480
AdC	180,57	51,156,867	138,609	38,486,575
Total	743,232	211,819,882	587,314	163,796,885

I: inoculated with *C. personatum*; S: drought stressed; C: control.

The high quality reads from the 454 GS FLX Titanium platform were then used for clustering and *de novo* assembly according to the genotype of origin, resulting in 17,912 unigenes (singletons and contigs) from *A. stenosperma* and 21,714 from *A. duranensis* (Table 2) with an average index of 83.8% of accepted reads for all the four libraries. The difference in the number of unigenes generated for the two species can be attributed to the fact that *A. duranensis* reads represent leaf and root transcriptomes, whilst *A. stenosperma* is solely composed from leaf tissues. The number of assembled unigenes found in this study including both species (39,626) was comparable to those reported for other legumes such as pigeonpea [19,30], mungbean [29], lentil [27] and chickpea [28] also obtained using the 454 GS FLX Titanium platform. 

**Table 2:** Total number of unigenes and genome coverage for each species

	***A. stenosperma***	***A. duranensis ***	**Average number for both species**	**Total number in both species**
Number of high quality reads	310,430	276,884	293,657	587,314
Number of reads after clustering	253,134	238, 456	245,795	491,590
*Percentage of accepted reads (%)	81.54	86.12	83.83	-
Number of unigenes	17,912	21,714	19,813	39,626
Number of contigs	7,723	12,792	10,257	20,257
Average contigs length (bp)	457	494	475.5	-
Average number of reads per contig	33	19	26	-
Contig genome coverage (Mbp)	8.18	10.72	9.45	18.91

*The index is calculated as the percentage of the total number of high–quality sequence reads per number of reads after clustering.

**Figure 2:**
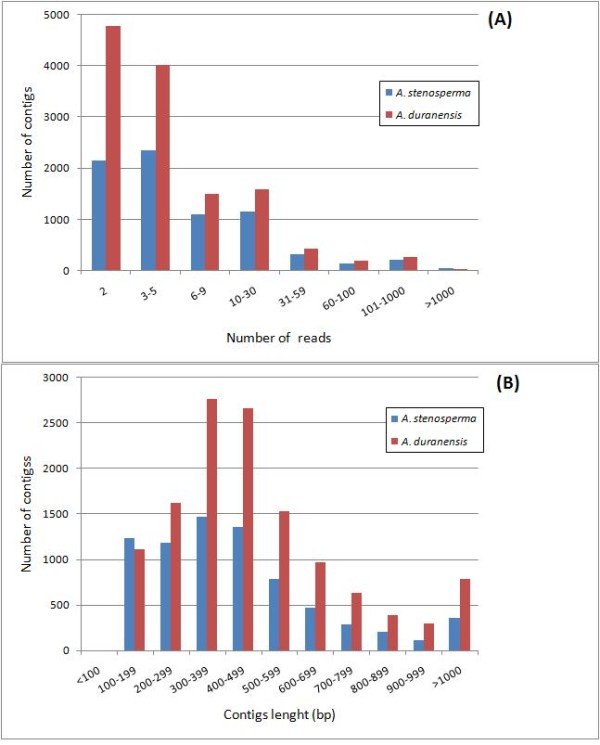
Distribution of contigs by number of reads (A) and length (B).

After removing the singletons, we produced 7,723 high confidence consensus sequences (contigs) for *A. stenosperma* and 12,792 for *A. duranensis*, with each contig being built from, on average, a relatively high number of reads (33 for *A. stenosperma* and 19 for *A. duranensis*) (Table 2). The number of reads/contig and their length distribution are shown in Figure 2 (A and B). The majority of the contigs were assembled from 2 to 5 reads, with 90% of them containing less than 30 reads for both species (Figure 2). The average length of the contigs was 457 bp for *A. stenosperma* and 494 bp for *A. duranensis,* with 27% and 36% of them larger than 500 bp respectively (Figure 2).

The genome coverage of contigs from *A. stenosperma* was 8.18 Mbp and *A. duranensis* 10.72 Mbp (Table 2), which make just under 1% of the estimated 1,260 Mbp size of a typical diploid *Arachis* species [35]. Sequence data from this study can be found for each species in the Sequence Read Archive (SRA) at the NCBI (*A. duranensis* - SRA047273.1; *A. stenosperma* - SRA047258.1). The derived contigs for each species and their most significant match against the nr database of GenBank (E value < e-7) is available in additional files (Additional files 1, 2) and at NCBI in Transcriptome Shotgun Assembly (TSA) (*A. duranensis* - JR332677 -JR344253 and *A. stenosperma* - JR326556 - JR332676).

### Sequence annotation and gene ontology

Only the high confidence consensus sequences (contigs) of *A. stenosperma* and *A. duranensis* were compared against the NCBI non-redundant protein sequence database (nr) for each species using BLASTX [36] in order to annotate known proteins/genes (Additional files 1, 2). A relatively high rate of contigs, 52.3% from *A. stenosperma* and 58.5% from *A. duranensis*, could be assigned to putative orthologs of genes involved in various pathways and cellular processes, when compared to other legumes without a completely annotated reference genome sequence [27,29]. Over 27% of the overall transcripts in both, *A. stenosperma* and *A. duranensis* showed homology in BLASTX to 14 legume species (Additional files 1 and 2). From these, over 60% of *A. stenosperma* and *A. duranensis* transcripts showed homology to *Glycine max,* followed by *Medicago* spp. (18%). Only 2.9% of *A. stenosperma* transcripts showed homology to *A. hypogaea*, whilst 6.5% showed homology to *A. duranensis*. This data reflects the greater number of ESTs available for these two legumes in comparison with *Arachis* spp. and also the closeness of *A. duranensis* to the cultivated tetraploid *A. hypogaea*[37].

**Figure 3:**
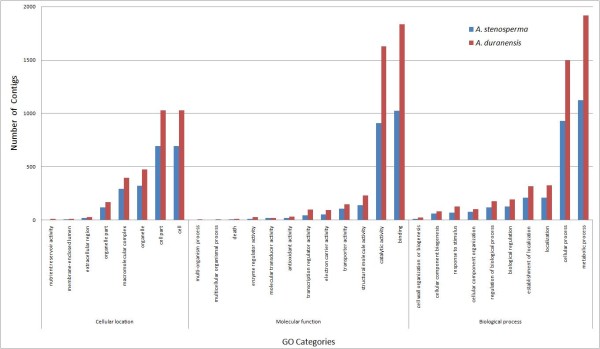
**GO Annotation analysis for contigs from *A. stenosperma *and *A. duranensis.*** Gene Ontology (GO) classification of the predicted *A. stenosperma* (blue) and *A. duranensis* (red) ORFs according to cellular location, molecular function and biological process using Blast2GO with e-10 cutoff.

For functional annotation, Blast2GO [38] was applied to classify contigs at superfamily, family and subfamily levels, to predict the occurrence of functional domains, repeats and important sites, and to include GO (Gene Ontology) terms to the protein signatures. From the 7,723 contigs in *A. stenosperma,* 96% (7,391) could be assigned to one or more GO annotation category, with 2,925 (39%) attributed to a biological process, 2,144 (29%) to a cellular component and 3,338 to a molecular function (45%) (Figure 3). Likewise, in *A. duranensis*, an equally high amount (12,024 contigs; 94%) of the 12,792 contigs could be appointed to GO annotation categories, with 4,752 (39%) identified as belonging to a biological process, 3,135 (26%) to a cellular component and 5,937 (49%) to a molecular function (Figure 3). 

The assignments made to the molecular function ontology was very similar for both species (Figure 3), with a large proportion of the sequences in catalytic (12–15%) and binding activities (14–17%), whilst under the biological process ontology a large proportion fell into metabolic process (15–17%) and cellular process (13%). Additionally, in *A. stenosperma*, including transcripts from fungi inoculated leaves, 68 sequences were identified in the GO subcategory response to stimulus which included peroxidases, catalases, chitinases, glycosinases and serine/threonine kinases, whilst in *A. duranensis*, including transcripts from leaves and roots submitted to water limited stress, 126 sequences were in this category with highlight to those sequences related to osmotic stress and water deprivation (Figure 3).

### Transcription factors

**Figure 4:**
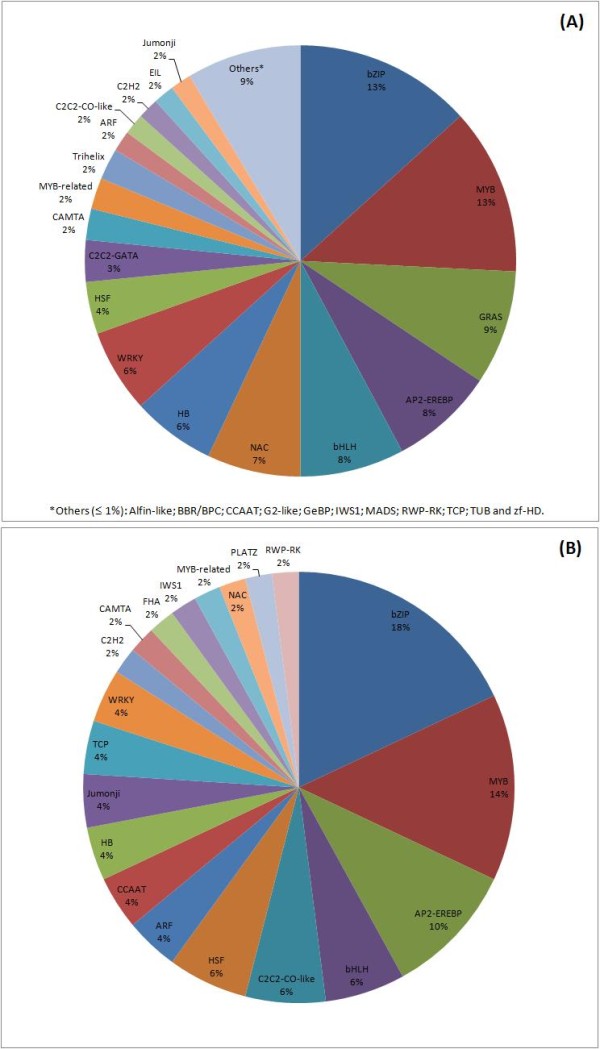
**Distribution of contigs of *A. Duranensis *(A) and *A. stenosperma *(B) by transcription factor (TF) families.** Transcription factors (TFs) identified by conserved domain annotation BLASTX with e-7 cutoff.

Transcription factors (TFs) constituted up to 1% of the total high confidence consensus sequences in both species studied, and were classified in TF families by sequence comparison to known transcription factor gene families at a Plant TF public database [39] (Figure 4). In this study, all TF *A. duranensis* transcripts were classified in 25 families that play important roles in eliciting stress responses such as bZIP (13%), MYB (13%), NAC (7%), bHLH and AP2-EREB (8%) and WRK (6%), the latter being the most highly represented (Figure 4A). In *A. stenosperma,* a slightly different distribution of the TFs in 20 families was observed with bZIp (18%), MYB (14%), AP2-EREB (10%), bHLH (6%) and WRK (4%), also being the most represented (Figure 4B). 

### Expression profile of RGAs and FIDEL

**Figure 5:**
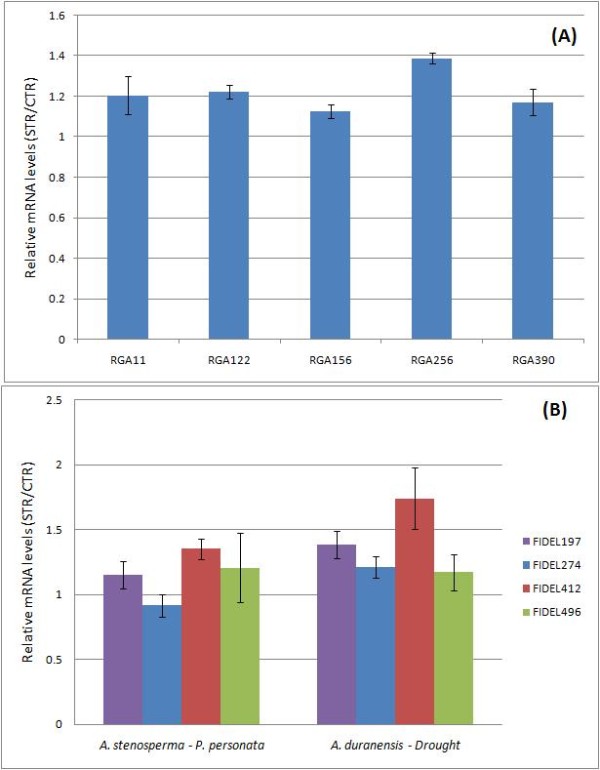
**Relative mRNA levels produced by five NBS-LRR sequences in *A. stenosperma *leaves inoculated with *C. personatum *(A) and by four FIDEL sequences in water limited stressed *A. duranensis *roots (B).** Normalization of expression was performed using as references the 60S gene for *A. stenosperma* and the actin gene for *A. duranensis* samples. Bars represent the standard error of the mean of two biological replicates for each sample.

The largest class of known plant disease resistance (R) proteins includes those that contain a nucleotide binding site and leucine-rich repeat domains (NBS-LRR proteins). NBS-LRR proteins may recognize the presence of a pathogen directly or indirectly [40]. A total of 48 homologs of *Arabidopsis* NBS encoding genes was identified in *A. stenosperma* according to previous methodology [41]*,* of which five representatives were selected for further expression analysis (Additional file 3). Those five genes were analyzed by qRT-PCR, using cDNA from *C. personatum* inoculated plants and the respective controls as template, RGA primers described in Table 3, and 60S as the reference gene, according to [42]. Relative quantification of transcripts showed that all five RGAs were up regulated in fungi-challenged plants in comparison to the control, with RGAs 256, 122 and 11 showing the biggest differences in expression levels (Figure 5A). 

**Table 3:** NBS-LRR and FIDEL sequences used for expression analysis using qRT-PCR

**Gene/Sequence abbreviation**	**Primer sequence Forward/Reverse**	**Amplicon size (bp)**	**PCR efficiency (%)**	**Regression coefficient R**^**2**^
RGA11	GGCAAAGGTGATGCTACCTG/	160	95.86	0.996
	TTCCCAAGGAAGTTGATCCT			
RGA122	TTGCACCAACTTCTGGTTTG/	156	94.41	0.979
	CAATGATATGCCCCGTCTCT			
RGA156	TCTAGAAAGCGAGGAGCTCAA/	157	96.09	0.991
	TGGATTGACTGGGATATGCTC			
RGA256	AGCTTCACGGTGAGGAAGAA/	178	93.09	0.981
	ATTCCGTGTTGCAGAACCTC			
RGA390	CAGAACAACGAGAACAATCG/	150	104.76	0.999
	TTACCGTCATCCGAAACTTG			
FIDEL197	TGGCTACATCCCATCCTCTC/	167	113.25	0.994
	GGGATTGAATGACTGTGACG			
FIDEL274	AGCTTTTTGCTTCGGAACAG/	161	95.92	0.903
	GGGTCACAGCCAAGACTCAT			
FIDEL412	GAGTTAAACGCCAGCTTTGG/	183	95.25	0.999
	CGGCGTTGGAAAGTAGACAT			
FIDEL496	GCAAGCTCGTGTAGGGTTTC/	157	110.58	0.997
	GCGTTAGTGACAGACGCAAA			
60S*	TGGAGTGAGAGGTGCATTTG/	155	102.36	0.998
	TCTTTTGACGACCAGGGAAC			
ACT1*	TGGTCTCGGTTTCCTGAGTT/	114	94.13	0.995
	AATACCACTCCAAAGCAAACG			

* Primer pair previously described [42].

NBS-LRR and FIDEL sequences differentially expressed *in silico* between *A. stenosperma* inoculated with *C. personatum* or *A. duranensis* drought-stressed and their respective controls.

Retroelements constitute the major part of repetitive DNA of a number of animal and plant genomes [43]. The long terminal repeat (LTR) retrotransposon-FIDEL constitutes a significant part of *Arachis* tetraploid and diploid genomes [20]. FIDEL-related sequences were found to be expressed in both species studied with a surprisingly high frequency. For *A. duranensis,* 0.23% of the high quality sequence reads, and 37 of the 12,792 (0.29%) contigs were FIDEL or FIDEL-related. For *A. stenosperma,* 1.3% of the high quality sequence reads, and 87 of the 7,465 (1.16%) contigs were FIDEL or FIDEL-related. *In silico* analysis indicated that most of these contigs were up regulated in response to the biotic/abiotic stresses. Four FIDEL-related contigs (Table 3) were chosen for analysis by qRT-PCR using cDNA from *A. stenosperma* leaves and *A. duranensis* roots, as template, and actin or 60S as reference genes.

We found that, with the exception of FIDEL274 in *A. stenosperma*/*C. personatum* samples, all representatives of this retroelement showed an increased expression in both species under biotic and abiotic stress (Table 3; Figure 5B). It is also interesting to note that, the levels of induction of the four FIDEL sequences were slightly higher in *A. duranensis* submitted to water limited stress than in *A. stenosperma* under fungi inoculation (Figure 5). The sequence FIDEL 412 showed the highest difference in expression levels between stressed and non-stressed plants with 1.74-fold expression ratio (Figure 5B).

### SSR identification

The discovery of EST-SSRs in the transcriptome of both species, *A. duranensis* and *A. stenosperma*, was performed based on the analysis from assembled contig templates. A total of 2,884 distinct SSR loci were identified, and 1,463 primer pairs were designed for *A. duranensis* and 862 for *A. stenosperma* corresponding to 11 and 10% of total contigs, respectively. Table 4 shows the information regarding the primer design and the frequency of different repeat types. Overall, the most abundant SSR type was tri-nucleotides (57%) followed by tetra/penta (20%) and di-nucleotides (12%). More details about the primers are provided in Additional file 4. Thirty–one EST-SSRs identified had been identified in a previous study [17] and were already mapped in the A-genome mapping population (*A. stenosperma* X *A. duranensis*) [11,19] (Additional file 4). The 2,324 primer pairs designed were submitted to ePCR [44] and 584 amplified in both species. Of these, 214 showed to be polymorphic for *A. duranensis* and *A. stenosperma*, which are the parents of a RIL mapping population. Some of these newly developed markers will be included in the saturated linkage map that is being constructed for the A-genome of *Arachis* using this RIL population. 

**Table 4:** **Frequency and repeat type of SSRs in *A. duranensis *and *A. stenosperma *transcripts**

**SSR mining**	***A. duranensis***	***A. stenosperma***	**Total**
Total sequences analyzed	13,132	8,480	21,612
Total sequences with repeats	1,701	1,183	2,884
Total primers designed	1,463	862	2,324
SSR in repeat types			
Di-nucleotide	157	115	272
Tri-nucleotide	838	488	1,326
Tetra,Penta-nucleotide	315	161	476
Hexa-nucleotide	153	98	251
SSR polymorphism			
Amplified in other parent*	376	208	584
Polymorphic ^§^	142	72	214
Percentage of polymorphic primers	-	-	36.6

* SSRs identified in one parent also amplified in the other parent.

§ Polymorphic SSRs identified from the set of primers amplifying in both species.

## Discussion

Transcriptome sequences are a valuable resource, especially for species without a completely sequenced genome, such as peanut. They accelerate gene discovery, provide an asset for molecular markers development and allow expression analysis and evolutionary genome dynamics studies. In the present study, Next Generation Sequencing (NGS) enabled the generation of large numbers of sequence reads in a rapid and cost-effective manner, and enabled the development of genomic resources for the exploitation of the stress resistances harbored by two wild diploid relatives of peanut.

Some recent studies have indicated that short reads from 454 GS 20 and GS FLX can effectively be used to characterize gene regions in a number of less studied species, including some tropical legumes [26,28-30,45,46]. In the present study, the average read length for both species was of 280 bp, which allowed estimated genome coverage of up to 163 Mbp of high quality reads for both diploid *Arachis* genomes studied in a single sequencing run. In comparison with other studies in legumes, a relatively small number of singletons were produced (8,922 for *A. duranensis* and 10,189 for *A. stenosperma*), furthermore the average length and number of reads per contig assembled was comparatively high (475.5 bp and 26 reads/contig) (Table 2) [27,29,47]. This may in part be due to very stringent quality and assembly parameters used, which also may partly explain that only 5% of the contigs produced in this study (1,012) failed to show significant functional annotation.

The lack of a complete sequenced and annotated reference genome makes it very difficult to estimate the genome coverage obtained in this study for both species analyzed. However, if we take as comparison other diploid legume genomes which have already been completely sequenced and assume the same number of genes, as for *Medicago truncatula* (38,835) and *Lotus japonicus* (42,395), we could suggest that up to 54% of the *A. duranensis* (21,714) and 44% of *A. stenosperma* (17,912) unigenes were covered in our work. However, it is also important to be aware that more than one contig or singleton can be originated from a single gene due to either non-overlapping sequence reads or high levels of sequence error in a single read [27].

Transcription factors (TFs) are of special interest due to their role in controlling plant developmental processes and responses to environmental conditions, including functions of key importance to agronomic performance [24]. They have an essential role in the signal transduction networks that leads from the perception of stress signals to the expression of stress-responsive genes, and, as opposed to most structural genes, tend to control multiple pathway steps within a transcriptional cascade [25]. Therefore, TFs are expected to be excellent candidates for modifying complex traits in crop plants, with TF-based technologies likely to be a prominent part of the next generation of successful biotechnology crops [48,49]. In the present study, 1% of the transcripts were identified as transcription factors (TFs). Their overall distribution among the various known TF protein families was compatible with previous studies in other legumes such as soybean, chickpea, pigeonpea and cultivated peanut [4,28,30,50,51], with bZIP, MYB, NAC, bHLH, AP2-EREBP and WRKY highly represented in both *A. duranensis* and *A. stenosperma* transcripts.

The most expressed TF family was the basic leucine zipper (bZIP)-type TF protein, which comprise regulators of many central developmental and physiological processes and abiotic and biotic stress responses [52]. Among other reports, this TF has been associated with water deficit-response in the relatively drought resistant tepary bean (*Phaseolus acutifolius)*[53] and to abscisic acid (ABA)-regulated gene expression required for the dehydration-response in *Arabidopsis*[54]. Likewise, this TF family was the most expressed in *A. duranensis* plants subjected to gradual water limited stress (18%), suggesting a role of this family in this relatively drought tolerant species. The bZIP TF family was also the most expressed TF in *A. stenosperma* leaves subjected to *C. personatum* (18%), and has already been described as involved in defense response to other host-fungi interactions, such as to the stripe rust via the ethylene/methyl jasmonate -dependent signal transduction pathways in wheat [55], and to regulate the expression of some stress-responsive genes such as the PR-1 and Glutathione S-Transferase in *Arabidopsis*[56].

The second most highly expressed TF family in drought imposed *A. duranensis* plants (12%) and fungi infected *A. stenosperma* leaves (14%) was the MYB family, which has been described to act through the ABA signaling cascade to regulate stomatal movement and therefore water loss regulation, and disease resistance in *Arabidopsis* and rice [57,58]. Likewise, the plant specific NAC transcription family was showed to be highly expressed in *A. duranensis* (10%) and to a lesser extent in *A. stenosperma* (2%). NAC proteins function has been previously described in potato and *Brassica napus* under fungal infection [59,60] and to significantly increase drought tolerance in soybean and chickpea [61,62].

Dehydration-responsive element binding (DREB) proteins a subgroup of the AP2/EREBP, have an important role in plant response and adaptation to abiotic stresses [63]. In this study, they constituted 7% of the TFs in *A. duranensis* plants subjected to water limited stress. A previous study with transgenic peanut plants over expressing *DREB1A* showed that the changes in the antioxidative machinery in these transgenic plants under water-limiting conditions played no causative role in improved transpiration efficiency [5,64,65]. Nonetheless, different DREB homologues have shown to play different roles in increasing tolerance to cold, salt and drought in different plant species, and have been extensively studied in *Arabidopsis*, rice and soybean being correlated to increased dehydration tolerance in these species [66-69]. An additional consideration is that recent studies indicate that function of central regulators as NAC, WRKY, and zinc finger proteins may be modulated by mechanisms such as small RNA (miRNA)-mediated posttranscriptional silencing, reactive oxygen species signaling and epigenetic processes such as DNA methylation and posttranslational modifications of histones [70]. This suggests that a more comprehensive elucidation of the role and dynamics of drought and defense responsive TFs in plants may be required.

Retroelements, particularly the long terminal repeat (LTR) retrotransposons, constitute the major part of repetitive DNA of plant genomes. Some of these elements seem to be constitutively expressed and others are silent and can be activated upon certain stress signals such as tissue culture, ionizing irradiation, wounding or poliploidization. As a matter of fact, data from the whole genome sequencing of several eukaryotes strongly suggests that, far from being circumstantial, the activity of transposable elements plays an extremely important role in the plasticity and regulation of host gene functions [71]. The mechanisms of how stress induces the activity of an element are not completely clarified, but it has been shown that most expression features of Tnt1, a Solanaceae retrotransposon, can be deduced from the structure of its regulatory regions, located in the LTR that contains several *cis*-acting elements, which are similar to well characterized motifs involved in activation of defense genes, whilst the Tnt1A G-box-like sequence is related to the typical ABA-responsive (ABRE) sequences and is identical to the MYC recognition sequence present in many drought-inducible genes [71,72].

In the present study, many transcripts from both species were identified as having similarity to retroelements. Therefore, we studied in more detail FIDEL, the only fully characterized Ty3-gypsy retrotransposon described in allotetraploid peanut (*A. hypogaea*) and its putative diploid ancestors *A. duranensis* (A genome) and *A. ipaënsis* (B genome) [20]. Using qRT-PCR analysis, we observed that FIDEL showed an increased expression ratio in both, *A. duranensis* roots subjected to gradual water limited stress and *A. stenosperma* leaves inoculated with fungus, when compared to non-challenged plants. In tobacco and other Solanaceae, drought stress and fungi infection have been described as triggering independent mechanisms of plant defense response and activation of transcription factors and retroelements [71,73]. In our study, we observed that both biotic and abiotic stresses induced FIDEL or FIDEL-related sequences. However, if the induction of FIDEL represents an activation of some specific FIDEL sequences, FIDEL harboring regions or some more specific response is not known.

Plants, in response to pathogen effectors, have co-evolved specific cytoplasm resistance R protein receptors which recognize individual pathogen effector molecular signatures and activate a second line of defense known as effector-triggered immunity (ETI) [74], also previously known as gene-for-gene or race-specific resistance. In contrast to non-specific response (PAMP-triggered immunity-PTI), which will occur in all members of a particular plant species, ETI operates at the intra-specific level, with resistant genotypes possessing the necessary R gene allele [75]. Conservation of motifs within R genes, such as those present within nucleotide-binding site leucine rich repeat domains, have facilitated their characterization in diverse plant taxa. Putative R genes or Resistance Gene Analogs (RGAs) are commonly clustered, as a result of duplication events occurring under diversifying selection. In *Arachis*, a previous investigation on RGAs content in a number of wild species [41] showed that from the 78 NBS sequences identified, most fall within legume-specific clades, some of which appear to have undergone extensive copy number expansions. In the present study, all five RGA sequences showed an increase on expression under *C. personatum* inoculation, when compared to the basal expression in the control samples. This was hardly unexpected, as proteins encoded by disease resistance (R) genes, are mostly constitutively expressed in resistant genotypes, mediating specific molecular recognition of pathogenic microorganisms and triggering signaling cascades that activate defense reactions [76,77]. A broader characterization of the transcriptional response of a suite of defense genes following stimulation of these R-genes, (i.e. kinases, peroxidases, transcription factors, NPR1) [78], and the defense pathways that they trigger is being conducted via Illumina deep sequencing. This will allow a better understanding of their contribution to the overall resistance response of *A. stenosperma* to *C. personatum*.

The transcriptome databank produced in this study enabled the development of 2325 SSR primer pairs of which 214 showed to be polymorphic between the two species. These new markers will enrich the current reference AA diploid Arachis map [19] and other Arachis tetraploid maps under construction. In addition, these EST-SSRs markers exhibit potential advantages when compared to SSRs located in non-transcribed regions due to generally more consistent efficiency of amplification, and enhanced cross-species transferability [27].

The development of new SSRs is of special interest in Arachis because these are still the markers of choice in this genus, due to the difficulties in the application of SNPs markers on the cultivated tetraploid species. Therefore, these new markers will contribute to enrich existing genetic maps, generate more informative genetic and genomic tools and enable the identification of orthologous genes through genome synteny analysis [15].

## Conclusions

The use of NGS for transcriptome sequencing of species without a complete reference genome is an effective approach for gene discovery and identification of transcripts involved in specific biological processes. The present work constitutes the largest unigene dataset for *A. stenosperma* and the second for *A. duranensis*, providing an insight into genomic architecture of these species and also creating a scaffold of transcribed sequences which will help to elucidate genes involved in biological processes such as fungi and drought- related response genes.

## Methods

### Plant material and library construction

Seeds of *A. stenosperma* (V10309) and *A. duranensis* (K7988) were obtained from the Brazilian *Arachis* Germplasm Collection, and maintained at Embrapa Genetic Resources and Biotechnology (Brasilia–DF, Brazil). For fungi bioassays, two month old plants of *A. stenosperma* were inoculated with a 50,000 spores⁄ml suspension in 0.5% Tween 20. Plant leaves were collected at 24, 48 and 72 hours after inoculation (HAI) and from non-inoculated controls, as described in our previous work [42], and immediately frozen on liquid nitrogen for RNA extraction.

For gradual water limited stress experiments, *A. duranensis* three months-old plants were equally divided into two groups of 33 individuals each: one group was subjected to a gradual water limited stress (STR), whilst the control group (CTR) was kept at approximately 70% of field capacity. Daily individual transpiration rate of STR and CTR plants was estimated gravimetrically and no more than 10 g of water loss per day was allowed in STR plants. Normalized Transpiration Ratio (NTR) was calculated between individual transpiration of STR and the mean transpiration of CTR plants, essentially as described by [79]. Leaves and roots were collected at distinct stages of the progressive water deficit (decreasing NTRs: 0.76; 0.73; 0.57; 0.43 and 0.40) and immediately frozen on liquid nitrogen to proceed RNA extraction.

Total RNA was extracted from 250 mg of plant material as previously described [42]. RNA integrity was checked by gel electrophoresis and quantified using Nanodrop ND-1000 (Thermo Scientific, Waltham, USA). To construct four bulked libraries, equal amounts of total RNA for inoculated *A. stenosperma* (leaves collected at 24, 48 and 72 HAI) and stressed *A. duranensis* (leaves and roots from all NTR points) were pooled separately from their respective non-treated controls and used for mRNA isolation. For the cDNA libraries construction and sequencing, services of CD-Genomics (http://www.cd-genomics.com) were used employing the Creator SMART cDNA library construction kit (Clontech Laboratories, California, USA) and Roche 454 GS-FLX System with Titanium chemistry.

### Sequence processing and assembly

Raw 454 data was pre-processed using est2assembly [80] for contaminant removal (non-coding RNA and plastidial sequences), quality trimming and adaptor trimming and poly-A removal. Transcript clustering was carried out using MIRA [81].

### Similarity search and functional annotation

Functional annotation of the cluster consensi was performed by sequence similarity searches using BLASTX program [36] against NCBI’s non-redundant sequence database. InterProScan [38] was employed to perform protein domain and motif searches. Gene ontology (GO) terms were assigned by Blast2GO [82].

For the identification of NBS encoding genes in *A. stenosperma*, predicted *Arabidopsis* NBS containing proteins identified as described in [41] were used as a BLAST database against which, all *A. stenosperma* contigs were used as query sequences in a BLASTX search. BLAST detected similarities were considered significant with E-values of 1e-7 or less (Additional file 3). Similarly, predicted FIDEL sequences identified in previous studies [20] were used as a BLAST database against which all *A. stenosperma* and *A. duranensis* contigs were used as query sequences in a BLASTX search (E value < 1e-7).

For the identification of TF families represented in this study, from the functional BLASTX annotation (value < 1e-7), all putative TF genes from both *Arachis* species were selected and, classified according to their respective TF family using the Plant TF database [39].

### SSRs identification

The program Mreps [83] was employed for the identification of simple sequence repeat (SSR) along the sequences. The parameters were set to identify perfect di- to hexa-nucleotide repeats with a minimum length of 12 bases. A series of custom-made PERL scripts were created to processes the potential SSR loci and to create flanking primers, based on Primer3 [84]. Electronic PCR was carried out using the PrimerMatch package [85] for the identification of primers amplifying in both species, and from these, the polymorphic set between *A. duranensis* and *A. stenosperma*.

### Expression analysis by qRT-PCR

Plant materials were obtained in new independent experiments carried out as described above.

After isolation and purification, total RNA of four samples (*A.stenosperma* leaves inoculated with *C. personatum* and *A. duranensis* roots stressed by water limitation and their respective non-treated controls) was digested with DNase (TURBO DNA-free™, Ambion, USA) and reverse-transcribed using SuperScript™ II RT and Anchored Oligo(dT)20 primer (Invitrogen, Carlsbad, CA, USA), as previously described [42]. For qRT-PCR, the Platinum® SYBR® Green qPCR Super Mix-UDG w/ROX kit (Invitrogen, Carlsbad, CA, USA) was used according to manufacturer's recommendations on a ABI 7300 Real-Time PCR System (Applied Biosystem Foster City, CA, USA). Two biological replicates for each of four samples were used for real-time PCR analysis, with each replicate representing a pool of five plants. Reactions were carried out using three technical replicates for each sample. Specific primer pairs were designed for five RGAs and four Fidel – related sequences (Table 3) with Primer3Plus software [84] and qRT-PCR cycling conditions were carried out with a final dissociation curve step, using previously described parameters [42]. Normalization of expression was performed using as references the 60S gene for *A. stenosperma* and the actin gene for *A. duranensis* samples [42]. All calculations for relative quantification, such as amplification efficiencies, correlation coefficients R2 values and relative expression profile (comparative C*t* method) were performed using 7500 v.2.0.4 software (Applied Biosystem, Foster City, CA, USA).

## Competing interests

The authors declare that they have no competing interests.

## Authors’ contributions

PMG conceived the study, produced the RNA samples and drafted the manuscript. ACMB produced the RNA samples, the qRT-PCR data and contributed to the writing of the manuscript. CM produced the RNA samples and the qRT-PCR data. AM produced the RNA samples and the qRT-PCR data. GP performed the bioinformatics analysis. OBSJr performed the bioinformatics analysis. RT performed the bioinformatics analysis. SCMLB performed the drought and fungi bioassays and contributed to the writing of the manuscript. ACGA performed the drought and fungi bioassays and contributed to the writing of the manuscript. MCM performed the SSR analysis. DJB conceived the study, performed the SSR analysis and contributed to the writing of the manuscript. All authors read and approved the final manuscript.

## Supplementary Material

Additional file 1 **Consensus sequences of assembled contigs of *A. stenosperma *and bioinformatic annotation (BLASTX)**. The data represents the consensus sequences of 7,468 assembled contigs of *A. stenosperma* generated as a result of *de novo* assembly and the three best BLASTX scoring hits obtained as a result of comparison of *A. stenosperma* contig set against nr database of GenBank at an E value < e-7. Click here for file

Additional file 2 **Consensus sequences of assembled contigs of *A. duranensis *and bioinformatic annotation (BLASTX)**. The data represents the consensus sequences of 12,791 assembled contigs of *A. duranensis* generated as a result of *de novo* assembly and the three best BLAST X scoring hits obtained as a result of comparison of *A. duranensis* contig set against nr databse of GenBank at an E value < e-7. Click here for file

Additional file 3 **Bioinformatic annotation (BLASTX) of *A. stenosperma *contig set against *Arabidopsis thaliana *genome.** This file contains the BLAST X results obtained as a result of comparison of *A. stenosperma* contig set against *A. thaliana* predicted NBS containing proteins. Click here for file

Additional file 4 **Sequence information of all SSR primer pairs designed using MREPS.***A stenosperma * and *A. duranensis* primer pairs identified and designed using MREPS and other information (sequence information, orientation, sequence length, expected product length, Tm and SSR motif length). Click here for file

## References

[B1] The FAO Statistical Database (FAOSTAT) http://faostat.fao.org/site/567/default.aspx

[B2] Tillman BL Stalker HT Vollmann J Rajcan I Prohens J, Nuez F, Carena MJ Peanut-oil crops Handbook of Plant Breeding. Volume 4 2010 New York: Springer 287 315

[B3] Payton P Kottapalli KR Rowland D Faircloth W Guo BZ Burow M Puppala N Gallo M Gene expression profiling in peanut using high density oligonucleotide microarrays BMC Genomics 2009 10 265 10.1186/1471-2164-10-265 19523230 PMC2703657

[B4] Kumar K Kirti P Differential gene expression in *Arachis diogoi* upon interaction with peanut late leaf spot pathogen, *Phaeoisariopsis personata* and characterization of a pathogen induced cyclophilin Plant Mol Biol 2011 75 497 513 10.1007/s11103-011-9747-3 21298396

[B5] Bhatnagar-Mathur P Devi MJ Reddy DS Lavanya M Vadez V Serraj R Yamaguchi-Shinozaki K Sharma KK Stress-inducible expression of At DREB1A in transgenic peanut (*Arachis hypogaea* L.) increases transpiration efficiency under water-limiting conditions Plant Cell Rep 2007 26 2071 2082 10.1007/s00299-007-0406-8 17653723

[B6] Nelson SC Simpson CE Starr JL Resistance to *Meloidogyne arenaria* in *Arachis* spp. germoplasm Suppl J NEMATOL 1989 21 654 660 PMC2618985 19287667

[B7] Freitas FO Moretzsohn MC Valls JFM Genetic variability of Brazilian Indian landraces of *Arachis hypogaea* L Genet Mol Res 2007 6 675 684 18050088

[B8] Bechara MD Moretzsohn MC Palmieri DA Monteiro JP Bacci M Martins J Valls JF Lopes CR Gimenes MA Phylogenetic relationships in genus Arachis based on ITS and 5.8S rDNA sequences BMC Plant Biol 2010 10 255 255 10.1186/1471-2229-10-255 21092103 PMC3095334

[B9] Guimarães P Brasileiro A Proite K de Araújo A Leal-Bertioli S Pic-Taylor A da Silva F Morgante C Ribeiro S Bertioli D A study of gene expression in the nematode resistant wild peanut relative, *Arachis stenosperma*, in response to challenge with *Meloidogyne arenaria* Trop Plant Biol 2010 3 183 192 10.1007/s12042-010-9056-z

[B10] Leal-Bertioli SCM De Farias MP Silva Pedro IT, Guimaraes PM, Brasileiro ACM, Bertioli DJ, De Araujo ACG: Ultrastructure of the initial interaction of *Puccinia arachidis* and *Cercosporidium personatum* with leaves of *Arachis hypogaea* and *Arachis stenosperma* J Phytopathol 2010 158 792 796 10.1111/j.1439-0434.2010.01704.x

[B11] Leal-Bertioli SCM Jose ACVF Alves-Freitas DMT Moretzsohn MC Guimarães PM Nielen S Vidigal BS Pereira RW Pike J Favero AP Identification of candidate genome regions controlling disease resistance in *Arachis* BMC Plant Biol 2009 9 112 10.1186/1471-2229-9-112 19698131 PMC2739205

[B12] Kaprovicas G Gregory WC Taxonomia del gênero *Arachis* (Leguminosae) Bonplantia 1994 8 1 186

[B13] Valls JFM Simpson CE Kerridge PH B Taxonomy, natural distribution, and attributes of *Arachis* Biology and agronomy of forage Arachis 1994 Cali: CIAT 1 18

[B14] Varshney R Bertioli D Moretzsohn M Vadez V Krishnamurthy L Aruna R Nigam S Moss B Seetha K Ravi K The first SSR-based genetic linkage map for cultivated groundnut (*Arachis hypogaea* L.) TAG Theor Appl Genet 2009 118 729 739 10.1007/s00122-008-0933-x 19048225

[B15] Bertioli D Moretzsohn M Madsen LH Sandal N Leal-Bertioli S Guimaraes P Hougaard BK Fredslund J Schauser L Nielsen AM An analysis of synteny of *Arachis* with *Lotus* and *Medicago* sheds new light on the structure, stability and evolution of legume genomes BMC Genomics 2009 10 45 10.1186/1471-2164-10-45 19166586 PMC2656529

[B16] Guimarães PM Garsmeur O Proite K Leal-Bertioli SC Seijo G Chaine C Bertioli DJ D’Hont A BAC libraries construction from the ancestral diploid genomes of the allotetraploid cultivated peanut BMC Plant Biol 2008 8 14 10.1186/1471-2229-8-14 18230166 PMC2254395

[B17] Proite K Leal-Bertioli SC Bertioli DJ Moretzsohn MC da Silva FR Martins NF Guimaraes PM ESTs from a wild *Arachis* species for gene discovery and marker development BMC Plant Biol 2007 7 7 10.1186/1471-2229-7-7 17302987 PMC1808460

[B18] Pandey MK Monyo E Ozias-Akins P Liang X Guimarães P Nigam SN Upadhyaya HD Janila P Zhang X Guo B Advances in Arachis genomics for peanut improvement Biotechnol Adv 2011 30 639 651 22094114 10.1016/j.biotechadv.2011.11.001

[B19] Moretzsohn MC Leoi L Proite K Guimarães PM Leal-Bertioli SCM Gimenes MA Martins WS Valls JFM Grattapaglia D Bertioli DJ A microsatellite-based, gene-rich linkage map for the AA genome of *Arachis* (Fabaceae) Theor Appl Genet 2005 111 1060 1071 10.1007/s00122-005-0028-x 16088397

[B20] Nielen S Campos-Fonseca F Leal-Bertioli S Guimarães P Seijo G Town C Arrial R Bertioli D FIDEL - a retrovirus-like retrotransposon and its distinct evolutionary histories in the A- and B-genome components of cultivated peanut Chromosome Res 2010 18 227 246 10.1007/s10577-009-9109-z 20127167 PMC2844528

[B21] Nielen S Vidigal B Leal-Bertioli S Ratnaparkhe M Paterson A Garsmeur O D’Hont A Guimarães P Bertioli D Matita, a new retroelement from peanut: characterization and evolutionary context in the light of the Arachis A B genome divergence Mol Genet Genomics 2011 287 21 38 22120641 10.1007/s00438-011-0656-6

[B22] Luo M Dang P Bausher MG Holbrook CC Lee RD Lynch RE Guo BZ Identification of transcripts involved in resistance responses to leaf spot disease caused by *Cercosporidium personatum* in peanut (*Arachis hypogaea*) Phytopathology 2005 95 381 387 10.1094/PHYTO-95-0381 18943040

[B23] Ranganayakulu G Chandraobulreddy P Thippeswamy M Veeranagamallaiah G Sudhakar C Identification of drought stress-responsive genes from drought-tolerant groundnut cultivar (*Arachis hypogaea* L. cv K-134) through analysis of subtracted expressed sequence tags Acta Physiologiae Plantarum 2012 34 361 377 10.1007/s11738-011-0835-4

[B24] Libault M Joshi T Benedito VA Xu D Udvardi MK Stacey G Legume transcription factor genes: what makes legumes so special? Plant Physiol 2009 151 991 1001 10.1104/pp.109.144105 19726573 PMC2773095

[B25] Udvardi MK Kakar K Wandrey M Montanari O Murray J Andriankaja A Zhang J-Y Benedito V Hofer JMI Chueng F Town CD Legume transcription factors: global regulators of plant development and response to the environment Plant Physiol 2007 144 538 549 10.1104/pp.107.098061 17556517 PMC1914172

[B26] Parchman T Geist K Grahnen J Benkman C Buerkle CA Transcriptome sequencing in an ecologically important tree species: assembly, annotation, and marker discovery BMC Genomics 2010 11 180 10.1186/1471-2164-11-180 20233449 PMC2851599

[B27] Kaur S Cogan N Pembleton L Shinozuka M Savin K Materne M Forster J Transcriptome sequencing of lentil based on second-generation technology permits large-scale unigene assembly and SSR marker discovery BMC Genomics 2011 12 265 10.1186/1471-2164-12-265 21609489 PMC3113791

[B28] Hiremath PJ Farmer A Cannon SB Woodward J Kudapa H Tuteja R Kumar A BhanuPrakash A Mulaosmanovic B Gujaria N Large-scale transcriptome analysis in chickpea (*Cicer arietinum* L.), an orphan legume crop of the semi-arid tropics of Asia and Africa Plant Biotechnol J 2011 9 922 931 10.1111/j.1467-7652.2011.00625.x 21615673 PMC3437486

[B29] Tangphatsornruang S Somta P Uthaipaisanwong P Chanprasert J Sangsrakru D Seehalak W Sommanas W Tragoonrung S Srinives P Characterization of microsatellites and gene contents from genome shotgun sequences of mungbean (*Vigna radiata* (L.) Wilczek) BMC Plant Biol 2009 9 137 10.1186/1471-2229-9-137 19930676 PMC2788553

[B30] Dutta S Kumawat G Singh B Gupta D Singh S Dogra V Gaikwad K Sharma T Raje R Bandhopadhya T Development of genic-SSR markers by deep transcriptome sequencing in pigeonpea [*Cajanus cajan* (L.) Millspaugh] BMC Plant Biol 2011 11 17 10.1186/1471-2229-11-17 21251263 PMC3036606

[B31] Weber APM Weber KL Carr K Wilkerson C Ohlrogge JB Sampling the Arabidopsis transcriptome with massively parallel pyrosequencing Plant Physiol 2007 144 32 42 10.1104/pp.107.096677 17351049 PMC1913805

[B32] Zhang G Guo G Hu X Zhang Y Li Q Li R Zhuang R Lu Z He Z Fang X Deep RNA sequencing at single base-pair resolution reveals high complexity of the rice transcriptome Genome Res 2010 20 646 654 10.1101/gr.100677.109 20305017 PMC2860166

[B33] Martin J-F Pech N Meglecz E Ferreira S Costedoat C Dubut V Malausa T Gilles A Representativeness of microsatellite distributions in genomes, as revealed by 454 GS-FLX Titanium pyrosequencing BMC Genomics 2010 11 560 10.1186/1471-2164-11-560 20939885 PMC3091709

[B34] Hyten D Song Q Fickus E Quigley C Lim J-S Choi I-Y Hwang E-Y Pastor-Corrales M Cregan P High-throughput SNP discovery and assay development in common bean BMC Genomics 2010 11 475 10.1186/1471-2164-11-475 20712881 PMC3091671

[B35] Temsch E Greilhuber J Genome size in Arachis duranensis: a critical study Genome 2001 44 826 830 11681606

[B36] Altschul SF Madden TL Schaffer AA Zhang JH Zhang Z Miller W Lipman DJ Gapped BLAST and PSI-BLAST: a new generation of protein database search programs Nucleic Acids Res 1997 25 3389 3402 10.1093/nar/25.17.3389 9254694 PMC146917

[B37] Seijo JG Lavia GI Fernandez A Krapovickas A Ducasse D Moscone EA Physical mapping of the 5S and 18S–25S rRNA genes by FISH as evidence that *Arachis duranensis* and *A. ipaensis* are the wild diploid progenitors of *A. hypogaea* (Leguminosae) Am J Bot 2004 91 1294 1303 10.3732/ajb.91.9.1294 21652361

[B38] Zdobnov EM Apweiler R InterProScan - an integration platform for the signature-recognition methods in InterPro Bioinformatics 2001 17 847 848 10.1093/bioinformatics/17.9.847 11590104

[B39] PlnTFDB a Plant Transcription Factor Databasehttp://plntfdb.bio.uni-potsdam.de/v3.0

[B40] Meyers BC Kozik A Griego A Kuang H Michelmore RW Genome-wide analysis of NBS-LRR-encoding genes in Arabidopsis The Plant Cell Online 2003 15 809 834 10.1105/tpc.009308 PMC152331 12671079

[B41] Bertioli DJ Leal-Bertioli SCM Lion MB Santos VL Pappas G Cannon SB Guimaraes PM A large scale analysis of resistance gene homologues in Arachis Mol Genet Genomics 2003 270 34 45 10.1007/s00438-003-0893-4 12928866

[B42] Morgante C Guimaraes P Martins A Araujo A Leal-Bertioli S Bertioli D Brasileiro A Reference genes for quantitative reverse transcription-polymerase chain reaction expression studies in wild and cultivated peanut BMC Research Notes 2011 4 339 10.1186/1756-0500-4-339 21906295 PMC3180468

[B43] Bennetzen JL Ma J Devos KM Mechanisms of recent genome size variation in flowering plants Ann Bot 2005 95 127 132 10.1093/aob/mci008 15596462 PMC4246713

[B44] Schuler GD Sequence mapping by electronic PCR Genome Res 1997 7 541 550 9149949 10.1101/gr.7.5.541 PMC310656

[B45] Luo H Li Y Sun C Wu Q Song J Sun Y Steinmetz A Chen S Comparison of 454-ESTs from *Huperzia serrata* and *Phlegmariurus carinatus* reveals putative genes involved in lycopodium alkaloid biosynthesis and developmental regulation BMC Plant Biol 2010 10 209 10.1186/1471-2229-10-209 20854695 PMC2956558

[B46] Blanca J Canizares J Roig C Ziarsolo P Nuez F Pico B Transcriptome characterization and high throughput SSRs and SNPs discovery in *Cucurbita pepo* (Cucurbitaceae) BMC Genomics 2011 12 104 10.1186/1471-2164-12-104 21310031 PMC3049757

[B47] Dubey A Farmer A Schlueter J Cannon SB Abernathy B Tuteja R Woodward J Shah T Mulasmanovic B Kudapa H Defining the transcriptome assembly and its use for genome dynamics and transcriptome profiling studies in pigeonpea (*Cajanus cajan* L) DNA Res 2011 18 153 164 10.1093/dnares/dsr007 21565938 PMC3111231

[B48] Hussain SS Kayani MA Amjad M Transcription factors as tools to engineer enhanced drought stress tolerance in plants Biotechnol Prog 2011 27 297 306 10.1002/btpr.514 21302367

[B49] Century K Reuber TL Ratcliffe OJ Regulating the regulators: the future prospects for transcription-factor-based agricultural biotechnology products Plant Physiol 2008 147 20 29 10.1104/pp.108.117887 18443103 PMC2330319

[B50] Wang Z Libault M Joshi T Valliyodan B Nguyen H Xu D Stacey G Cheng J SoyDB: a knowledge database of soybean transcription factors BMC Plant Biol 2010 10 14 10.1186/1471-2229-10-14 20082720 PMC2826334

[B51] Govind G Vokkaliga ThammeGowda H Jayaker Kalaiarasi P Iyer D Muthappa S Nese S Makarla U Identification and functional validation of a unique set of drought induced genes preferentially expressed in response to gradual water stress in peanut Mol Genet Genomics 2009 281 591 605 10.1007/s00438-009-0432-z 19224247 PMC2757612

[B52] Correa LGG Riano-Pachon DM Schrago CG Vicentini dos Santos R Mueller-Roeber B Vincentz M The role of bZip transcription factors in green plant evolution: adaptive features emerging from four founder genes PLoS One 2008 3 e2944 10.1371/journal.pone.0002944 18698409 PMC2492810

[B53] Rodriguez-Uribe L O’Connell MA A root-specific bZIP transcription factor is responsive to water deficit stress in tepary bean (*Phaseolus acutifolius*) and common bean (*P. vulgaris*) J Exp Bot 2006 57 1391 1398 10.1093/jxb/erj118 16531461

[B54] Uno Y Furihata T Abe H Yoshida R Shinozaki K Yamaguchi-Shinozaki K *Arabidopsis* basic leucine zipper transcription factors involved in an abscisic acid-dependent signal transduction pathway under drought and high-salinity conditions Proc Natl Acad Sci 2000 97 11632 11637 10.1073/pnas.190309197 11005831 PMC17252

[B55] Zhang Y Zhang G Xia N Wang X-J Huang L-L Kang Z-S Cloning and characterization of a bZIP transcription factor gene in wheat and its expression in response to stripe rust pathogen infection and abiotic stresses Physiol Mol Plant Pathol 2008 73 88 94 10.1016/j.pmpp.2009.02.002

[B56] Singh KB Foley RC Onate-Sanchez L Transcription factors in plant defense and stress responses Curr Opin Plant Biol 2002 5 430 436 10.1016/S1369-5266(02)00289-3 12183182

[B57] Yanhui C Xiaoyuan Y Kun H Meihua L Jigang L Zhaofeng G Zhiqiang L Yunfei Z Xiaoxiao W Xiaoming Q The MYB transcription factor superfamily of *Arabidopsis:* expression analysis and phylogenetic comparison with the rice MYB family Plant Mol Biol 2006 60 107 124 10.1007/s11103-005-2910-y 16463103

[B58] Dai X Xu Y Ma Q Xu W Wang T Xue Y Chong K Overexpression of an R1R2R3 MYB gene, OsMYB3R-2, increases tolerance to freezing, drought, and salt stress in transgenic Arabidopsis Plant Physiol 2007 143 1739 1751 10.1104/pp.106.094532 17293435 PMC1851822

[B59] Olsen AN Ernst HA Leggio LL Skriver K NAC transcription factors: structurally distinct, functionally diverse Trends Plant Sci 2005 10 79 87 10.1016/j.tplants.2004.12.010 15708345

[B60] Wang X Basnayake BMVS Zhang H Li G Li W Virk N Mengiste T Song F The *Arabidopsis* ATAF1, a NAC transcription factor, is a negative regulator of defense responses against necrotrophic fungal and bacterial pathogens Mol Plant Microbe Interact 2009 22 1227 1238 10.1094/MPMI-22-10-1227 19737096

[B61] Le DT Nishiyama R Watanabe Y Mochida K Yamaguchi-Shinozaki K Shinozaki K Tran L-SP Genome-wide survey and expression analysis of the plant-specific NAC transcription factor family in soybean during development and dehydration stress DNA Research 2011 1 14 10.1093/dnares/dsr015 PMC3158466 21685489

[B62] Peng H Cheng H-Y Yu X-W Shi Q-H Zhang H Li J-G Ma H Characterization of a chickpea (*Cicer arietinum* L.) NAC family gene, CarNAC5, which is both developmentally- and stress-regulated Plant Physiol Biochem 2009 47 1037 1045 10.1016/j.plaphy.2009.09.002 19800808

[B63] Yamaguchi-Shinozaki K Shinozaki K Transcriptional regulatory networks in cellular responses and tolerance to dehydration and cold stresses Annu Rev Plant Biol 2006 57 781 803 10.1146/annurev.arplant.57.032905.105444 16669782

[B64] Bhatnagar-Mathur P Devi MJ Vadez V Sharma KK Differential antioxidative responses in transgenic peanut bear no relationship to their superior transpiration efficiency under drought stress J Plant Physiol 2009 166 1207 1217 10.1016/j.jplph.2009.01.001 19201508

[B65] Devi MJ Bhatnagar-Mathur P Sharma KK Serraj R Anwar SY Vadez V Relationships between transpiration efficiency and its surrogate traits in the *rd29A:DREB1A* transgenic lines of groundnut J Agron Crop Sci 2011 197 272 283 10.1111/j.1439-037X.2011.00464.x

[B66] Zhou JL Wang XF Jiao YL Qin YH Liu XG He K Chen C Ma LG Wang J Xiong LZ Global genome expression analysis of rice in response to drought and high-salinity stresses in shoot, flag leaf, and panicle Plant Mol Biol 2007 63 591 608 10.1007/s11103-006-9111-1 17225073 PMC1805039

[B67] Zhao L Hu Y Chong K Wang T ARAG1, an ABA-responsive DREB gene, plays a role in seed germination and drought tolerance of rice Ann Bot 2010 105 401 409 10.1093/aob/mcp303 20100696 PMC2826253

[B68] Li X-P Tian A-G Luo G-Z Gong Z-Z Zhang J-S Chen S-Y Soybean DRE-binding transcription factors that are responsive to abiotic stresses TAG Theor Appl Genet 2005 110 1355 1362 10.1007/s00122-004-1867-6 15841365

[B69] Chen M Wang Q-Y Cheng X-G Xu Z-S Li L-C Ye X-G Xia L-Q Ma Y-Z GmDREB2, a soybean DRE-binding transcription factor, conferred drought and high-salt tolerance in transgenic plants Biochem Biophys Res Commun 2007 353 299 305 10.1016/j.bbrc.2006.12.027 17178106

[B70] Golldack D Luking I Yang O Plant tolerance to drought and salinity: stress regulating transcription factors and their functional significance in the cellular transcriptional network Plant Cell Rep 2011 30 1383 1391 10.1007/s00299-011-1068-0 21476089

[B71] Grandbastien MA Audeon C Bonnivard E Casacuberta JM Chalhoub B Costa APP Le QH Melayah D Petit M Poncet C Stress activation and genomic impact of Tnt1 retrotransposons in Solanaceae Cytogenet Genome Res 2005 110 229 241 10.1159/000084957 16093677

[B72] Shinozaki K Yamaguchi-Shinozaki K Gene networks involved in drought stress response and tolerance J Exp Bot 2007 58 221 227 17075077 10.1093/jxb/erl164

[B73] Capy P Gasperi G Biemont C Bazin C Stress and transposable elements: co-evolution or useful parasites? Heredity 2000 85 101 106 10.1046/j.1365-2540.2000.00751.x 11012710

[B74] Jones JDG Dangl JL The plant immune system Nature 2006 444 323 329 10.1038/nature05286 17108957

[B75] Xiao S Wang W Yang X Heine H Evolution of resistance genes in plants innate immunity of plants, animals, and humans Nucleic Acids and Molecular Biology. Volume 21 2008 Berlin: Springer 1 25

[B76] Dangl JL Jones JDG Plant pathogens and integrated defence responses to infection Nature 2001 411 826 833 10.1038/35081161 11459065

[B77] Hammond-Kosack KE Jones JD Resistance gene-dependent plant defense responses Plant Cell 1996 8 1773 1791 8914325 10.1105/tpc.8.10.1773 PMC161314

[B78] Eulgem T Weigman VJ Chang H-S McDowell JM Holub EB Glazebrook J Zhu T Dangl JL Gene expression signatures from three genetically separable resistance gene signaling pathways for downy mildew resistance Plant Physiol 2004 135 1129 1144 10.1104/pp.104.040444 15181204 PMC514145

[B79] Ray JD Sinclair TR Stomatal closure of maize hybrids in response to drying soil Crop Sci 1997 37 803 807 10.2135/cropsci1997.0011183X003700030018x

[B80] Papanicolaou A Stierli R Ffrench-Constant R Heckel D Next generation transcriptomes for next generation genomes using est2assembly BMC Bioinforma 2009 10 447 10.1186/1471-2105-10-447 PMC3087352 20034392

[B81] Chevreux B Pfisterer T Drescher B Driesel AJ Muller WEG Wetter T Suhai S Using the miraEST assembler for reliable and automated mRNA transcript assembly and SNP detection in sequenced ESTs Genome Res 2004 14 1147 1159 10.1101/gr.1917404 15140833 PMC419793

[B82] Gotz S Garcia-Gomez JM Terol J Williams TD Nagaraj SH Nueda MJ Robles M Talon M Dopazo J Conesa A High-throughput functional annotation and data mining with the Blast2GO suite Nucleic Acids Res 2008 36 3420 3435 10.1093/nar/gkn176 18445632 PMC2425479

[B83] Kolpakov R Bana G Kucherov G mreps: efficient and flexible detection of tandem repeats in DNA Nucleic Acids Res 2003 31 3672 3678 10.1093/nar/gkg617 12824391 PMC169196

[B84] Untergasser A Nijveen H Rao X Bisseling T Geurts R Leunissen JAM Primer3Plus, an enhanced web interface to Primer3 Nucleic Acids Res 2007 35 W71 W74 10.1093/nar/gkm306 17485472 PMC1933133

[B85] Primer Matchhttp://edwardslab.bmcb.georgetown.edu/software/primer_match.html

